# Research hotspots and trends of oculomotor nerve palsy from 2001 to 2021 based on web of science: A bibliometric analysis

**DOI:** 10.3389/fneur.2023.1112070

**Published:** 2023-02-22

**Authors:** Runze Wang, Yang Gao, ShanHong Wu, Xiaojun Cai, TianYang Yu, Liyuan Wang

**Affiliations:** ^1^Heilongjiang University of Chinese Medicine, Harbin, China; ^2^Endocrinology Department, Heilongjiang Academy of Sciences of Traditional Chinese Medicine, Harbin, China; ^3^Acupuncture Department, Second Affiliated Hospital of Heilongjiang University of Chinese Medicine, Harbin, China; ^4^Ophthalmology Department, First Affiliated Hospital of Heilongjiang University of Chinese Medicine, Harbin, China

**Keywords:** oculomotor nerve paralysis, bibliometric analysis, Citespace, VOSviewer, web of science

## Abstract

**Background:**

Oculomotor nerve palsy (ONP) is a clinically occurring neurological disorder. Due to the complex anatomy and long travel distances of the oculomotor nerve, the causes of ONP vary and manifest in various ways. With continued interest in this area, it has become necessary to conduct a bibliometric study in ONP. This work aims to synthesize and visually identify current research themes and future trends in ONP through a literature-based analysis.

**Method:**

Articles and reviews on ONP published from 2002 to 2021 were derived from the Web of Science Core Collection (WoSCC) database. We generated visual images and performed quantitative and qualitative analysis through an online bibliometric tool, Citespace and VOSviewer software.

**Results:**

A total of 1,205 published articles were included in this analysis. The annual number of this area's publications is showing an overall upward trend, with the number of citations increasing every year, reaching 2,698 by 2021. The United States (367) and Japan (116) dominated the list with the most numerous articles published. The University of California Los Angeles in the USA is the institution that published the highest number of articles (47). Engle EC (23) and the JOURNAL OF NEUROSURGERY (46) are the most influential authors and journals in this field. The co-occurrence network analysis divided the keywords into five main research themes, which mainly include clinical manifestations of ONP, aneurysms, cerebral neurological symptoms, diseases with ONP as a complication, and other neurological disorders.

**Conclusion:**

This study is the first comprehensive and systematic bibliometric analysis of the current state of global ONP research over the past 20 years. We organized current hotspots and expected trends and provided key information for exploring potential research frontiers in ONP.

## Introduction

The oculomotor nerve (ON) is the main nerve that innervates eye movements. Its damage may lead to typical manifestations such as ptosis, diplopia, unable to do eye movements, and diminished or absent pupillary light reflex (1–6). In an early study of 290 patients with oculomotor nerve palsy (ONP), results showed 23.1% of patients had unknown causes, 20.7% had vascular lesions, 16.2% had head trauma, 14.5% had other causes, 13.8% had aneurysms, and 11.7% had tumors (7). Singh's report indicated that the causes of ONP were congenital or acquired, congenital for 43%, trauma for 20%, inflammation for 13%, intracranial aneurysms for 7% in pediatric patients, while vascular disease, intracranial aneurysms and trauma were the most common causes among adult patients (8). At the same time, diabetes mellitus, painful ophthalmoplegia, and cerebral infarction are also the etiology of this disease (9–11). A 10-year cohort study of Koreans showed that the incidence of ONP increased annually from 2006 to 2015 and was more prevalent in older adults, with a significantly accelerated incidence after age 60, severely affecting patients' quality of life (12). Therefore, the number of studies on ONP has increased dramatically in the past few decades. However, studies on publication patterns, literature characteristics, and bibliometrics on ONP are scarce.

Bibliometrics is the science of qualitative and quantitative analysis of existing research fields through mathematics and statistics (13, 14). Bibliometrics can provide readers with a visual and intuitive image of research results, including the contributions and productivity of different countries, institutions, and journals, as well as the evolution of information about the creators, the priorities, and the dissemination channels of the field. It emerged as a solution to the dilemma of access to knowledge due to the enormous growth of information and offered the possibility of interdisciplinary collaboration, crossover and integration.

In this study, we conducted a bibliometric analysis of published ONP-related studies and reviews in the Web of Science Core Collection (WoSCC) database over the past 20 years. This study can assist researchers in obtaining a brief overview of current hotspots in this field to gain insight into future research trends, to improve the quality of ONP research.

## Materials and methods

### Data acquisition and search strategy

All data were screened from the Science Citation Index-expanded (SCI-E) of the WoSCC database on November 6, 2022. After repeated attempts in a limited range, we finally used the query as follows: TS = (Oculomotor nerve disease^*^ OR Oculomotor nerve disorder OR Oculomotor neuropathy OR Oculomotor nerve paralysis OR Oculomotor nerve palsy) with a publication date from 2001-01-01 to 2021-12-31. We limited the document type as “articles” and “reviews” and the language as “English.” Finally, a total of 1,205 records were retrieved for bibliometric analysis.

### Data extraction

To facilitate processing with the software, we exported all records as “full records and citation references” and stored them as plain text files. The basic information included authors' names, institution, country/region, corresponding authors, citations count, journal, and keywords. The H-index and the average citations per item (ACI) were extracted from the Web of Science (WoS) webpage by using the “Create Citation Report” function.

### Data analysis

Microsoft Excel 2019 are mainly used to summarize basic information such as the number of annual publications and the number of citations. The different country publications over the years and country collaboration analysis were using the bibliometric platform (https://bibliometric.com/).

Citespace is the most widely used knowledge mapping tool for co-occurrence and burst analysis of institutions, authors, and keywords (15). In the mapping created by Citespace (Version 6.1.R4), the number or frequency of different item types (countries, institutions, and authors) is indicated by the size of the nodes. At the same time, the color represents the year in which the item appeared, with darker colors representing later appearances. The thicker the line, the more frequent the association.

VOSviewer, also a bibliometric software, is better at knowledge clustering and visual analysis (16). Therefore, in this study, we used this software (Version 1.6.18) to generate corresponding graphs to perform co-authorship and co-citation analyses of authors, journals, and references.

## Result

### Overall publication performance

**Figure 1:**
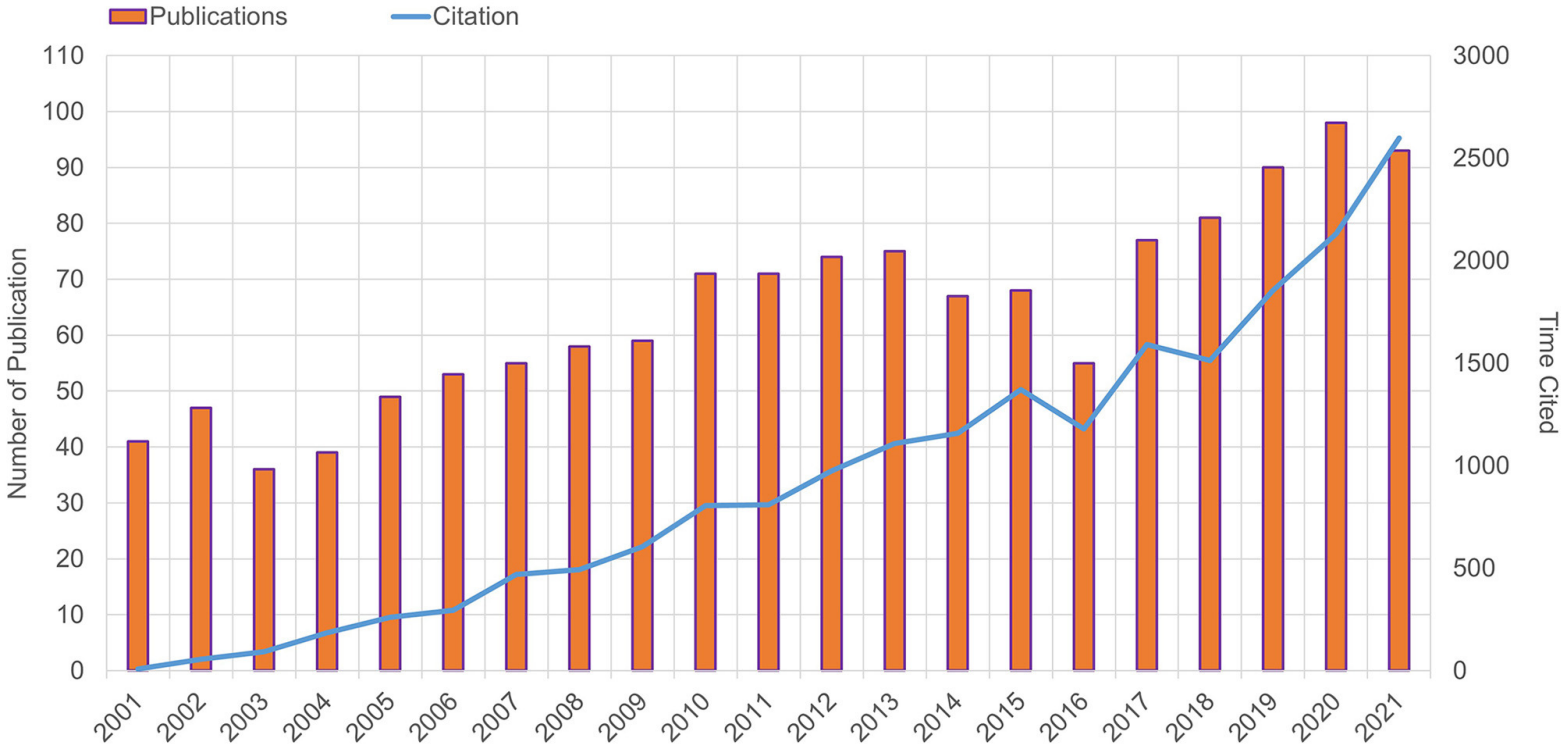
Publication and citation. Trends in publications and citations per year from 2001 to 2021.

The actual number of literatures in a field over a certain period can reveal the intensity of research in the discipline and give a general indication of its trends. As shown in Figure 1, the number of ongoing papers in the field has generally been upward over the last 20 years. The number of research publications was growing slowly from 2001 to 2009, with a significant increase in the annual production of publications in 2010, followed by a fluctuating upward trend over the next 11 years, which indicates that ONP has been a hot issue in the research field. Despite a small decline in the number of publications in 2021, it is still significant and considerable. According to our statistics, the total number of cited articles in this field was 15,001 (14,322 after removing self-citations), and the total number of citations was 22,625 (18,727 after removing self-citations), with an average of 17.95 citations per paper. The citation volume has been growing consistently and rapidly year over year, which indicates that ONP-related research has a certain influence.

### Countries and regions

**Figure 2:**
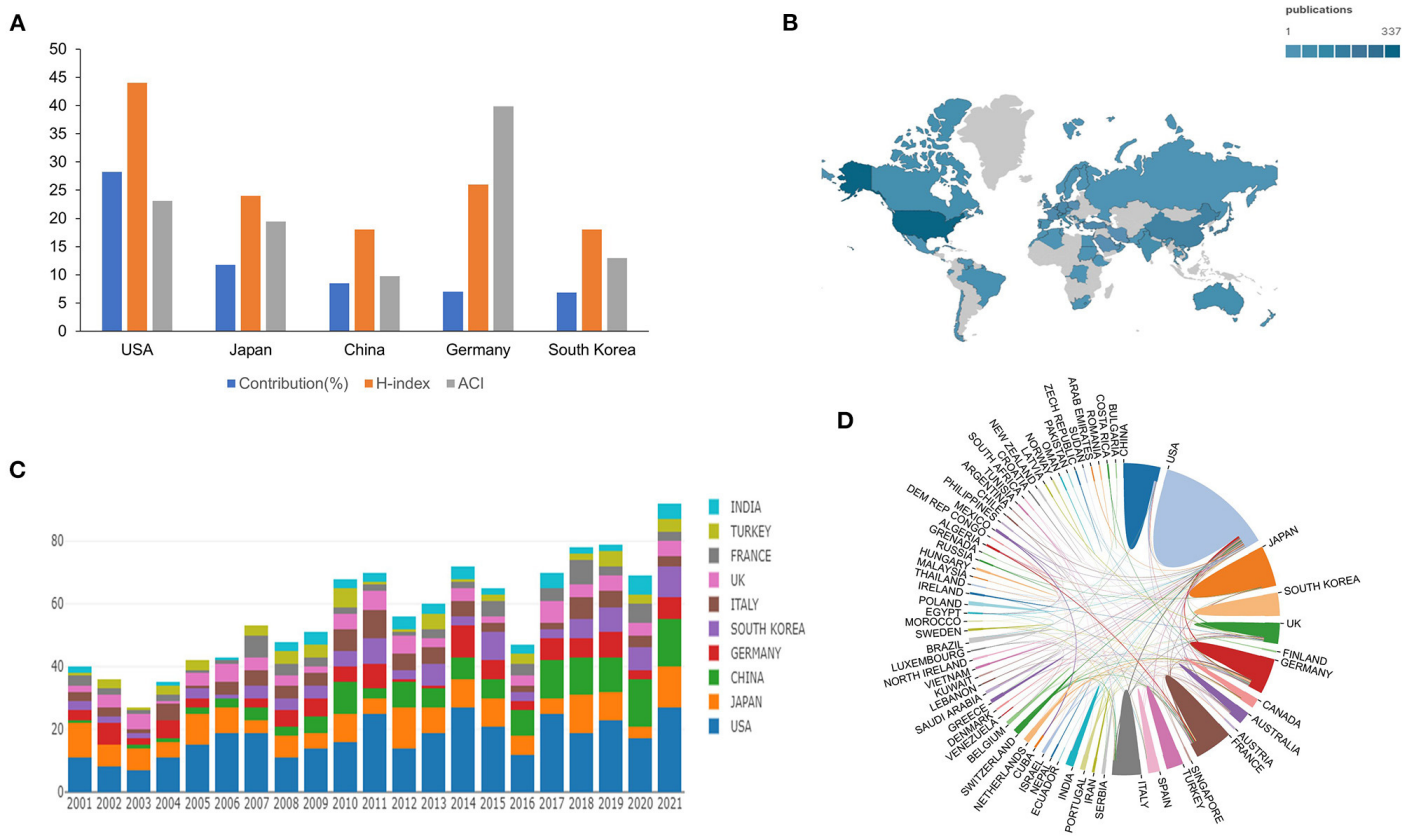
Countries and regions. **(A)** Top 5 most productive countries contribution. **(B)** Geographical distribution of countries in ONP. **(C)** The annual publication counts of the top 10 countries from 2001 to 2021. **(D)** Collaborative relationships among different countries.

The statistics indicated that altogether 1,205 documents got published from 75 different countries/regions. USA (340, 28.2%), Japan (142, 11.8%), China (102, 8.5%), Germany (84, 7.0%), and South Korea (83, 6.9%) were the top five countries in terms of the number of papers published showed in Figure 2A. The volume of articles issued by all countries was visualized in Figure 2B, while the annual volume was shown in Figure 2C. Figure 2D shows the network diagram of cooperation between countries. We analyzed the cooperation between countries and found that the USA cooperates most closely with Germany.

### Institutions

**Figure 3:**
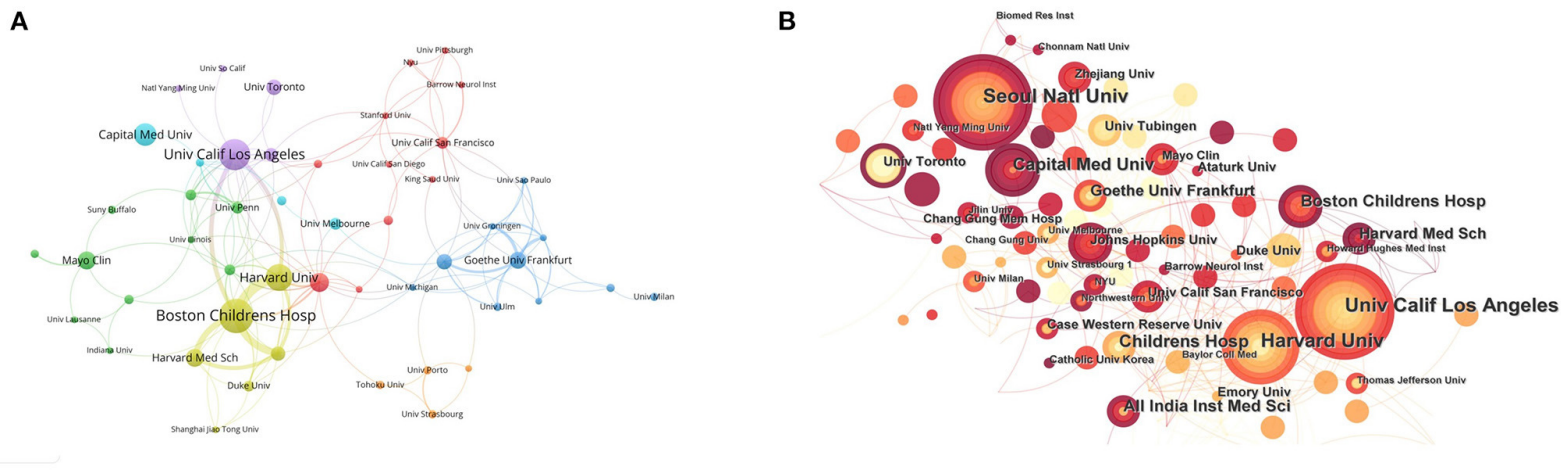
Institutions. **(A)** Co-occurrence analysis of the main institutions. The thicker the line between the institutions, the more frequently the two cooperate. **(B)** A Citespace visualization map of the cooperated institutions involved in ONP. The color of the node represents the time of the most recent ONP field paper published by this institution.

The top 15 most productive institutions are listed in Table 1. It shows that the University of California Los Angeles, Harvard University, Boston Children's Hospital, and Harvard Medical School have published more publications in the last two decades. Figure 3A visualizes the collaborative relationships among the institutions. From this figure, the University of California Los Angles collaborates more closely with Harvard University and Boston Children's Hospital. At the same time, Seoul National University has more publications but few collaborations with other institutions, which shows that the collaboration between different national institutions needs to be enhanced. Figure 3B reflects the timing of ONP-related studies published by each institution, which shows that Capital Medical School, Boston Children's Hospital, and Harvard Medical School have been very interested in this disease in the recent past.

**Table 1:** Top 15 most productive institutions between 2001 to 2021 on ONP.

**Rank**	**Insititution**	**Country**	**Number of documents**	**Total citations**	**Average citation**	**H-index**
1	University of California Los Angeles	USA	47	1,902	41.60	22
2	Harvard University	USA	46	1,765	40.35	20
3	Udice French Research Universities	France	39	1,884	49.95	18
4	Harvard Medical School	USA	37	1,372	39.54	18
5	Boston Children's Hospital	USA	29	1,301	47.97	17
6	Institut National de la Santé et de la Recherche Médicale	France	27	1,669	64.04	15
7	Seoul National University	South Korea	27	349	13.81	11
8	Capital Medical University	China	21	153	7.38	8
9	Johns Hopkins University	USA	21	608	29.05	12
10	Assistance Publique Hopitaux Paris Aphp	France	19	1,225	65.00	13
11	University of London	UK	18	806	44.83	9
12	Center National de la Recherche Scientifique Cnrs	France	17	1,672	100.88	14
13	Goethe University Frankfurt	Germany	17	740	44.82	16
14	University of Toronto	Canada	16	814	51.50	9
15	Mayo Clinic	USA	15	297	19.87	9

### Author and co-cited author

**Figure 4:**
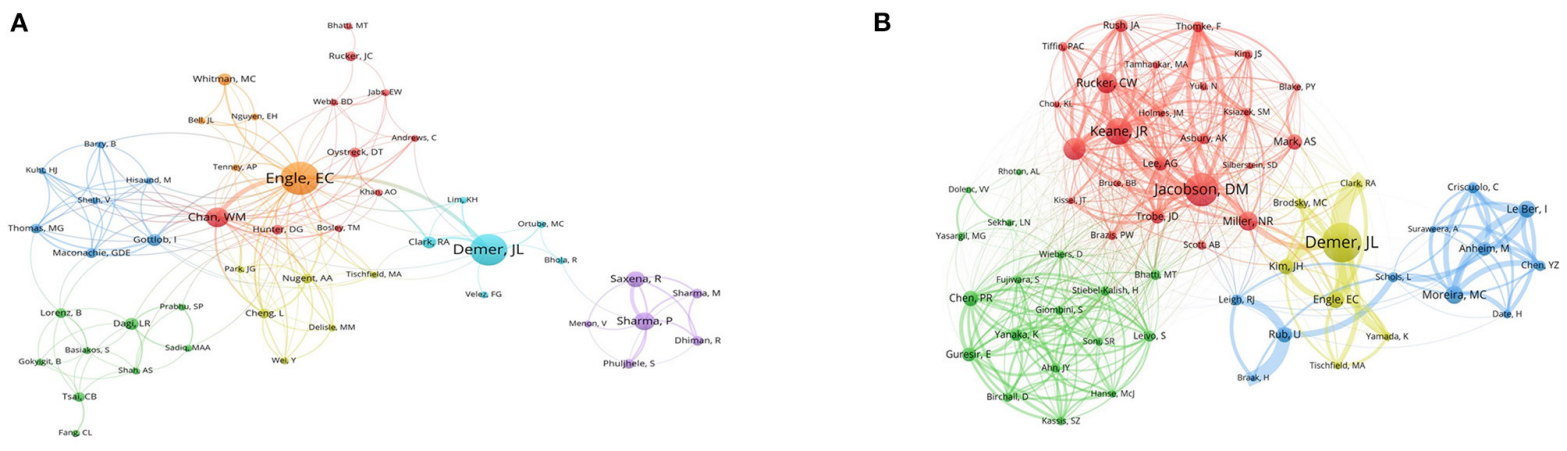
Author and co-cited author. **(A)** Network analysis of authors involved in ONP. **(B)** Network analysis of co-cited authors involved in ONP. The thicker the line between the institutions, the more frequently the two cooperate.

A total of 5,609 authors participated in the ONP study, with an average of 4.65 authors per article. Table 2 lists the top 15 authors regarding the number of publications, with Engle EC being the author with the most articles (*n* = 23), followed closely by Demer JL (*n* = 20). In Figure 4A, we visualized the collaborative relationships between authors and revealed that authors from the USA, France, and Germany collaborated more closely, but collaborative links between other countries remained low.

**Table 2:** The top 15 productive authors and co-cited authors on ONP research from 2001 to 2021.

**Rank**	**Author**						**Co-cited author**	
	**Name**	**Articles**	**Country**	**Total citations**	**Average citation**	**H-index**	**Name**	**Citations**
1	Engle EC	23	USA	1,282	59.09	16	Demer, JL	173
2	Demer JL	20	USA	1,012	52.95	15	Jacobson, DM	146
3	Hwang JM	14	South Korea	223	16.86	7	Keane, JR	118
4	Koenig M	14	France	1,344	99.14	12	Richards, BW	96
5	Kim JS	11	South Korea	148	13.55	5	Rucker, CW	90
6	Chan WM	10	USA	759	77.40	8	Miller, NR	83
7	Kim JH	10	South Korea	159	17.00	7	Moreira, MC	79
8	Rub U	10	Germany	457	47.60	10	Engle, EC	74
9	Schols L	8	Germany	656	82.63	7	Le Ber, I	74
10	Anheim M	7	France	328	48.43	7	Kim, JH	68
11	Gottlob I	7	UK	625	90.14	5	Rub, U	68
12	Moreira MC	7	France	1,063	153.14	7	Chen, PR	67
13	Oh SY	7	South Korea	44	6.57	5	Mark, AS	67
14	Sharma P	7	India	73	11.29	4	Anheim, M	61
15	Sharpe JA	7	Canada	66	10.71	5	Guresir, E	61

A total of 18,416 authors were cited in 1,205 publications, and we have compiled the information of the top 15 cited authors in Table 2. As can be seen from this table, three of the authors with more than 100 citations are Demer JL (*n* = 173), Jacobson DM (*n* = 146), and Keane JR (*n* = 118). After filtering out the authors with more than 30 citations, we used VOSviewer to visualize the contribution of these authors to the field in Figure 4B. As shown in this figure, Miller NR collaborated with the largest number of authors, while Demer JL had the most comprehensive impact.

### Journals

**Figure 5:**
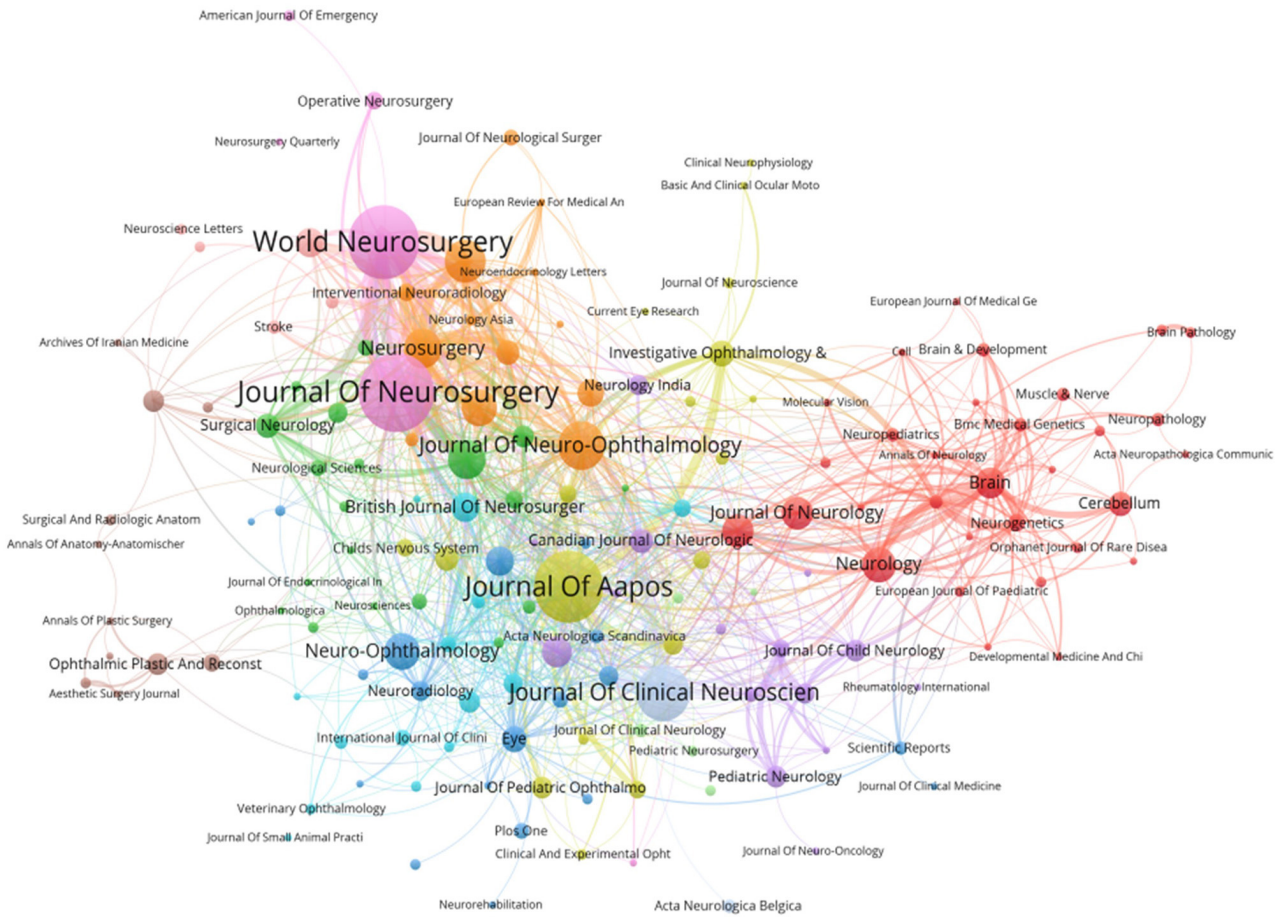
Journals. Visualized analysis of co-occurrence of journals built with VOSviewer.

The top 15 journals with the most publications are listed in Table 3, which shows that *Journal of Neurosurgery* has published the highest number of documents (*n* = 46), followed by *World Neurosurgery* (*n* = 42). The highest impact factor is *Brain*, which also has the highest number of citations and average citations, indicating its considerable impact in this field. According to the Journal Citation Report 2021, only 46.7% are located in Q1 and Q2 among the top 15 involved journals. Figure 5 visualizes the journal co-citation, revealing that *Journal of Neuro-Ophthalmology*'s publications are cited by more journals, while *Neurosurgery* is the one with the highest overall weight.

**Table 3:** The top 15 journals regarding the number of publications on ONP.

**Rank**	**Journal**	**Articles**	**Citations**	**Average citation**	**H-index**	**IF (2021)**	**QCR**	**5-year IF**
1	Journal of neurosurgery	46	744	16.39	17	5.408	Q1	5.266
2	World neurosurgery	42	313	7.9	10	2.21	Q4	2.336
3	Journal of AAPOS	41	517	13.2	14	1.325	Q4	1.52
4	Journal of clinical neuroscience	29	245	8.55	10	2.116	Q4	2.236
5	Journal of neuro ophthalmology	24	279	11.79	9	4.415	Q2	3.777
6	Acta neurochirurgica	20	164	8.4	10	2.816	Q3	2.603
7	Clinical neurology and neurosurgery	18	178	10	8	1.885	Q4	2.11
8	Neurosurgery	18	715	40.67	12	5.315	Q1	5.716
9	Neuro ophthalmology	16	17	1.13	3	/	Q4	/
10	Neurologia medico chirurgica	16	156	10.13	8	2.036	Q4	2.676
11	Neurology	15	478	32	11	12.258	Q1	11.786
12	Journal of neurology	14	282	20.21	7	6.682	Q1	6.174
13	Journal of the neurological sciences	14	200	14.43	7	4.553	Q2	3.861
14	Brain	13	977	75.85	13	15.255	Q1	16.173
15	Internal medicine	13	70	5.54	5	1.282	Q4	1.26

### Co-cited references

**Figure 6:**
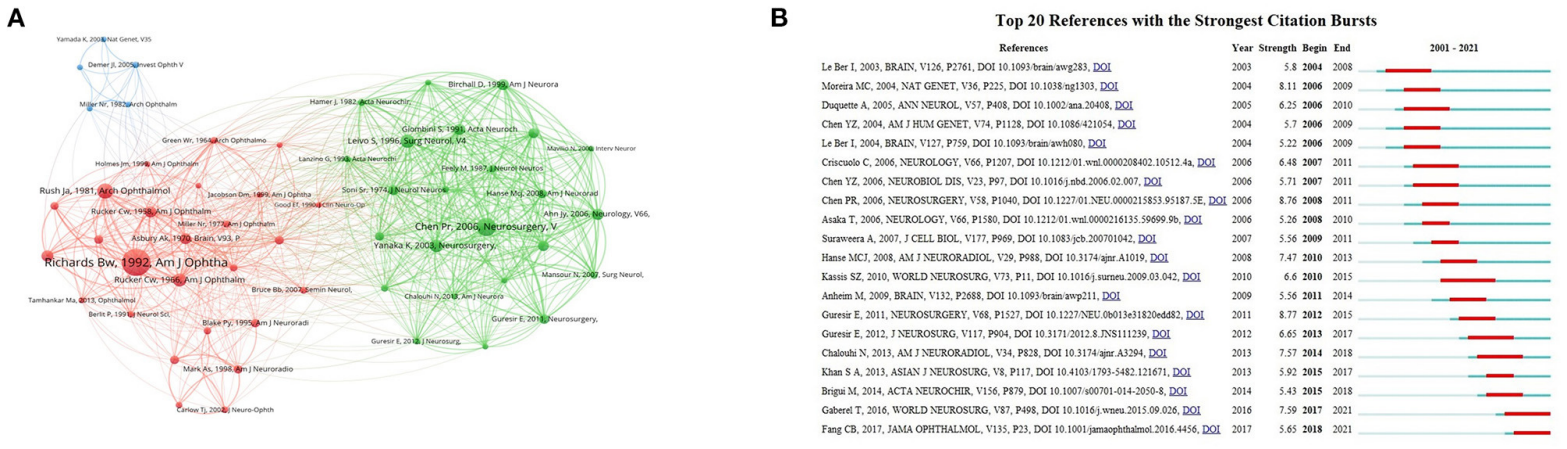
Co-cited references. **(A)** Network map of co-cited references on ONP. **(B)** The top 20 references with highest burst values on ONP from 2001 to 2021. The solid blue line represents the length of time the keyword appeared, and the red portion in the middle indicates the time of the reference burst.

According to our statistics, 1,205 articles were cited by 24,720 pieces of literature. Table 4 lists the information of the top 10 cited articles. We used publications cited more than 20 times to form a co-citation network to show the citation relationships among articles. As shown in Figure 6A, the article published by Richards BW in 1992 in the *American Journal of Ophthalmology* is the most co-cited literature. This study has a positive relationship with other studies, which became the basis for many subsequent studies.

**Table 4:** Top 10 most cited references.

**Rank**	**Title**	**Citations**	**Year**	**Journal**	**First author**
1	Causes and prognosis in 4,278 cases of paralysis of the oculomotor, trochlear, and abducens cranial nerves	96	1992	American journal of ophthalmology	Richards BW
2	Outcome of oculomotor nerve palsy from posterior communicating artery aneurysms: Comparison of clipping and coiling	65	2006	Neurosurgery	Chen PR
3	Paralysis of cranial nerves III, IV, and VI. Cause and prognosis in 1,000 cases	54	1981	Archives of ophthalmology	Rush JA
4	Early surgery improves the cure of aneurysm-induced oculomotor palsy	48	1996	Surgical neurology	Leivo S
5	The causes of paralysis of the third, fourth and sixth cranial nerves	47	1966	American journal of ophthalmology	Rucker CW
6	Small unruptured cerebral aneurysms presenting with oculomotor nerve palsy	46	2003	Neurosurgery	Yanaka K
7	Acquired palsy of the oculomotor, trochlear and abducens nerves	41	1996	Eye	Tiffin PAC
8	Clipping vs. coiling of posterior communicating artery aneurysms with third nerve palsy	40	2006	Neurology	Ahn JY
9	Paralysis of the third, fourth and sixth cranial nerves	40	1958	American journal of ophthalmology	Rucker CW
10	Oculomotor palsy in diabetes mellitus: a clinico-pathological study	39	1970	Brain	Asbury AK

Citation bursts can show the references that researchers follow in a certain time. As shown in Figure 6B, the top 20 references with the strongest citation bursts have burst values ranging from 5.22 to 8.77. Kassis (17), *World Neurosurg* has the longest burst, while Guresir (18), *Neurosurgery* has the highest burst value. The two most recent citation bursts are Gaberel (19), *World Neurosurg* and Fang et al. (20), *JAMA Ophthalmol*, with burst values of 7.59 and 5.65.

### Keywords and hotspots

**Figure 7:**
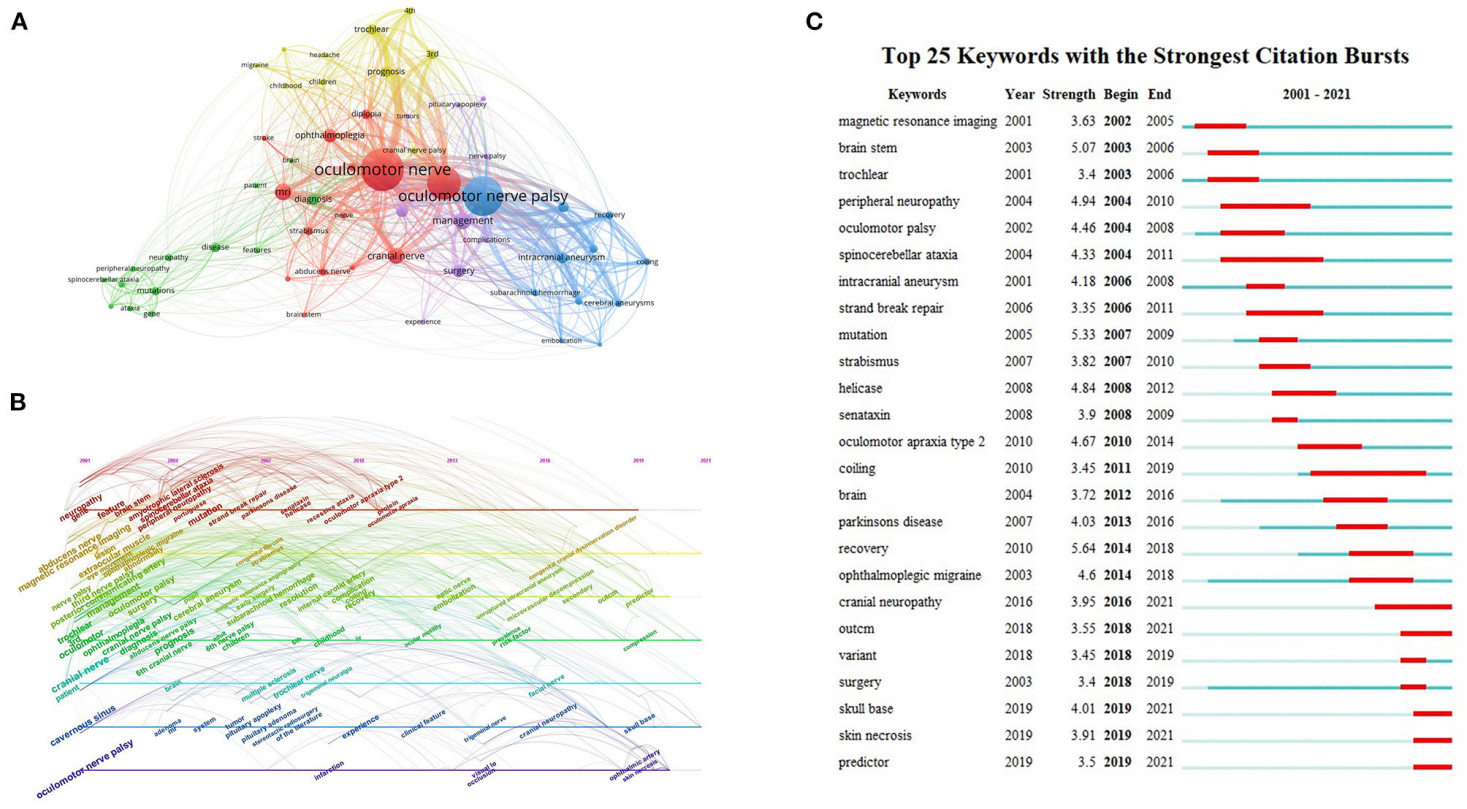
Keywords and hotspots. **(A)** Network map of ONP-related keywords co-citation. **(B)** Timeline map of ONP-related keywords appearance. **(C)** The top 25 keywords with the highest values of bursts in the ONP studies.

We extracted 4,401 keywords from 1,205 articles. Among these keywords, the top 10 most frequent keywords were oculomotor nerve (*n* = 301), oculomotor nerve palsy (*n* = 275), paralysis (*n* = 223), management (*n* = 96), MRI (*n* = 94), cranial nerve (*n* = 88), ophthalmoplegia (*n* = 71), surgery (*n* = 68), cavernous sinus (*n* = 61), and diagnosis (*n* = 58). After limiting the number of occurrences of keywords to more than 20, we obtained 57 keywords and plotted the co-occurrence network using VOSviewer in Figure 7A. The keywords were divided into 5 clusters. Cluster 1 (red) is mainly about clinical manifestations of ONP, such as ophthalmoplegia, diplopia, and ptosis. Cluster 2 (blue) is mainly related to aneurysms, such as intracranial and cerebral aneurysms. Cluster 3 (yellow) is mainly connected with other cerebral neurological symptoms and prognosis, such as trochlear, headache, and migraine. Cluster 4 (purple) is related to diseases with ONP as a complication, such as tumors, pituitary apoplexy, and cavernous sinus. Cluster 5 (green) is associated with other neurological disorders which may cause ONP, such as spinocerebellar ataxia, peripheral neuropathy, and gene mutations.

Using Citespace is possible to plot a timeline of keyword occurrences, and the timeline allows visualization of the stage hotspots and direction of ONP research. As shown in Figure 7B, researchers focused mainly on exploring the types of diseases associated with ONP symptoms in the first 10 years (2001–2010), with keywords including cavernous sinus, cerebral aneurysm, subarachnoid hemorrhage, pituitary adenoma, spinocerebellar ataxia, and amyotrophic lateral sclerosis. The research focus of 2011–2020 is tilted toward the treatment, prognosis, and management of ONP. The main keywords are as follows: clinical feature, predictor, risk factor, surgical technique, and complication.

Keyword bursts can further demonstrate the evolution of the field's hotspots and predict the next development trend. Figure 7C indicates that the burst value of ONP ranged from 3.35 to 5.64. “Recovery” is the keyword with the strongest emergent strength (5.64), and “coiling” has the longest outbreak time lasting a total of 8 years. Recent keywords include “skull base,” “skin necrosis,” and “predictor.”

## Discussion

ONP may lead to serious morbidity or life-threatening causes, often viewed as a diagnostic concern in clinical practice (20, 21). Most of these lesions causing ONP occurred in the subarachnoid space and cavernous sinus (22, 23). There are also some untypically causes, such as schwannoma, meningioma, and radiation, may also lead to ONP (21). The ON's local anatomy is complex, its course covers a long distance, and the distribution of the oculomotor nuclei in the midbrain is more dispersed, creating a higher risk of injury to this nerve (24). The main symptoms of ONP, such as ptosis, diplopia, and difficulty with eye movements, seriously affect patients' quality of life, making it of high research value. To our knowledge, there are no patterns, literature characteristics, or bibliometric analyses of ONP. In order to better understand the research hotspots and predict future research trends on ONP, we have conducted this manuscript to provide researchers with quick access to this information.

In this study, we presented an overall review of the literature in the field of ONP from 2001 to 2021. We provided a qualitative and quantitative overview of the allocation of studies over countries, authors, and journals. Moreover, we reviewed keywords and citations involved in the literature to clarify how they have developed and evolved. In terms of the number of papers, the number of articles on ONP has been on the rise in the past 20 years and peaking in 2020, with citations increasing year by year, indicating that researchers have devoted considerable effort to ONP in recent years, and this trend will continue in the future. An analysis of institutions and countries shows that the United States publishes the most articles on ONP and has the most productive research institutions. A total of 6 of the 15 most published institutions are in the United States. These institutions are ranked at the top of the H-index and ACI, which shows the influence of the US in the ONP field. However, the node centrality representing the United States is still low (0.12). Coupled with the relatively scattered locations of institutional co-occurrence maps, research countries and institutions with absolute dominance have not yet emerged. At the same time, academic exchanges and cooperation among institutions need to be further reinforced. It is interesting to note that although Germany ranks 4th in publication numbers, its ACI is the highest, reflecting the high recognition of ONP-related research in Germany.

The institution analysis shows that the University of California Los Angeles collaborates more closely with Harvard University and Boston Children's Hospital. After further searching, we found that the primary area of collaboration between these institutions is in the molecular biology of congenital fibrosis of the extraocular muscles (CFEOM). CFEOM is a rare hereditary strabismus characterized by extraocular muscle restriction and congenital ptosis, pathologically characterized by partial or complete damage to the oculomotor nucleus and the extraocular muscles innervated by the motor nerve (25, 26). The two most cited articles in the collaborative publications of these institutions reported mutations in the KIF21A gene that causes the CFEOM1 and mutations in the TUBB3 gene that causes the CFEOM3 (27, 28). In addition, neuroimaging studies of CFEOM caused by different mutations are also the focus of collaborative studies at these institutions (26, 29). These findings have contributed significantly to the development of the molecular genetics and clinical imaging manifestations of CFEOM. Author analysis shows Professor Engle EC, based at Harvard University, was the most productive author studying the molecular genetics of specific types of strabismus that manifest as damage to the ON, abducens nerve, and talocrural nerve. Engle first performed genetic analysis and two-point linkage mapping on two autosomal dominant CFEOM families in 1994 and localized the CFEOM1 gene to the 8 centimorgans of chromosome 12 between the D12S87 and D12S85 region (30). In subsequent studies, Engle's research team defined three CFEOM phenotypes due to different gene mutations by finer genetic mapping (31–33). They investigated the structural basis of oculomotor abnormalities in different types of CFEOM by magnetic resonance imaging (MRI) (26, 29). In a 2017 published article, she revealed a role for the ephrin bidirectional signaling pathway upstream of mutant alpha2 chimeric proteins in Duane retraction syndrome (DRS), which may contribute to the selective vulnerability of abducens motor neurons (34).

The journal analysis helps researchers access information on core and refereed journals in their subject areas (35). Table 3 shows that the *Journal of Neurosurgery* published the most articles, but the journal with the highest number of citations and average citations was the *Brain*. The majority of journals shown in this table have impact factors below 5, and only 5 journals are from Q1, suggesting that the quality of articles on ONP needs to be improved. However, it may also be related to researchers focusing more on diagnostic issues of ONP rather than information such as pathological mechanisms. The most cited article analyzes the causes and prognosis in 4,278 cases of paralysis of the oculomotor, trochlear, and abducens cranial nerves written by Richards BW in 1992 (*n* = 96) (36) published on the *American journal of ophthalmology*. Rush and Younge (7), Rucker (37), and Tiffin et al. (38) have conducted clinical studies on the analysis of the causes and prognosis of ONP-related diseases. The rest of the top 10 citations focus more on the effect of surgical treatment on ONP from artery aneurysms. Surgery focuses on preventing aneurysm rupture, re-rupture risk, and mitigating the nerve damage caused by compression (39). Surgical clipping, which immediately reduces the occupancy effect, has long been the preferred treatment for aneurysm-induced ONP (18, 40), but other research shown that endovascular treatment is more likely to result in better recovery of neurological function in patients (41–43). The prevailing explanation for coiling's ability to reduce or eliminate aneurysmal pulsatility is more beneficial to nerve recovery than surgical clipping that directly separates the oculomotor nerve from the adjacent aneurysm (39, 44, 45). Citation analysis of articles is likely to be affected by length-time effects (46), so we performed a burst analysis of the co-cited literature to interpret the results. A systematic review and meta-analysis of endovascular coiling and surgical clipping for ONP, published by Gaberel et al. (19) in *World Neurosurgery* in 2016, has recently seen a citation explosion. Another recent outbreak article focused on the etiology of ONP, and the characteristics of developing populations were published by Fang et al. (20) in 2017. As can be seen, most of the highly cited literature types are retrospective clinical studies that provide a reliable basis for the diagnosis, treatment, and prognosis of ONP.

Cooccurrence and burst analysis of keywords can reveal the hotspot distribution and the evolution of research trends in a field (47). As shown in Figure 7A, the keyword clustering of ONP is closely related to the clinical manifestations of the disease as well as its etiology. The pathogenesis of ONP is both congenital and acquired, and its common identifiable etiologies include cerebrovascular disease, aneurysm, trauma, brain tumor, and demyelinating disease (6, 48). The timeline provides us with a more accurate picture of the changes in each cluster over time. As can be seen in Figure 7B, “infarction,” “clinical feature,” “risk factor,” “ophthalmic artery,” and “skull base” have remained hot topics of research in ONP in the last decade, suggesting that risk and diagnostic studies of ONP still promising. As vascular ischemia is the most common clinical cause and risk factor of ONP in adults, the diagnosis should be based on the exclusion of patient history, symptoms, and radiological and biological information (49). Studies have shown that MRI 3D-CISS techniques can exclude aneurysm or tumor compression, local inflammation, and infection along the oculomotor nerve, which can help clarify the clinical diagnosis and thus avoid further extensive laboratory and imaging examinations (50). Notably, the keyword “skin necrosis” appears in recent outbreaks, which may be relevant to the new etiology of ONP highlighted by recent case reports. Sung et al. (51) reported a case of progressive ONP and patchy skin necrosis after calcium hydroxylapatite filler injection for nose augmentation in 2010, while Kwon et al. (52) reported a case of sudden onset of ONP and gradually occurring skin necrosis after filling soft tissue with hyaluronic acid injections in 2013, Bae et al. (53), Kim et al. (54), and Lucaciu et al. (55) reported similar cases in the past 5 years, reminding plastic surgeons that they should be aware of these risks. In Figure 7C, the keyword bursts show the evolution of ONP research hotspots and trends. The earliest outbreak of keywords was magnetic resonance imaging (MRI), which indicates that the diagnosis of diseases related to ONP, such as aneurysms and cerebrovascular diseases in the early years were mainly relied on MRI and other traditional imaging methods. MRI can also evaluate some genetic diseases that exhibit ONP, such as CFEOM and DRS. However, in recent years, the emergence of keywords, such as “predictors,” suggests that advances in molecular biology and gene localization studies have helped us gradually understand the underlying mechanisms of these genetic diseases. Moreover, it helped us to find predictive and diagnostic methods for these genetic diseases leading to ONP. The recent increase of the keyword “cranial neuropathy” suggests that researchers are becoming more concerned about ONP caused by congenital abnormal innervation of cranial nerves.

Several limitations of our study still need to be pointed out, although this does not impact our conclusions. First, this work only selected literature in the WOSCC database where the language was restricted to English, which may have led to incomplete results. Secondly, to ensure the accuracy and completeness of data trends over time, we only analyzed papers from 2001 to 2021, which may have led to the omission of some major articles published recently.

## Conclusion

In general, after analyzing 1,205 articles, we found that the U.S. institutions, authors, and the overall number of publications and citations are in the leading position, indicating the U.S.'s influence in the ONP field. Analysis of the co-cited literature shows that research on ONP in the last 20 years has focused on clinical diagnosis and the selection of surgical modalities. Clustering visualization of keywords revealed that the main causes of ONP are cerebrovascular disease, aneurysms, tumors, and demyelinating disease. However, burst analysis of keywords suggests we still need to focus on new causes of ONP and its complications during treatment.

This study provides a comprehensive and systematic overview of the current state of global ONP research over the past 20 years. The findings can provide researchers with key information in this field, such as current hotspots and expected trends, and guide them to explore potential research frontiers.

## Data availability statement

The original contributions presented in the study are included in the article/supplementary material, further inquiries can be directed to the corresponding authors.

## Author contributions

These articles were retrieved and downloaded by RW, YG, and SW. RW and YG were responsible for collecting data and visualizing the results. RW and XC wrote the first draft. TY and LW were provided guidance throughout the work, revising drafts, and approving the final manuscript. All authors contributed significantly to the final article.
